# Risk Factors of Diabetic Foot Amputation in Pakistani Type II Diabetes Individuals

**DOI:** 10.7759/cureus.4795

**Published:** 2019-06-01

**Authors:** Bhawna Nanwani, Prem Shankar, Ravi Kumar, Faizan Shaukat

**Affiliations:** 1 Internal Medicine, Jinnah Sindh Medical University, Karachi, PAK; 2 Internal Medicine, Dow Medical College, Karachi, PAK; 3 Internal Medicine, Chandka Medical College, Larkana, PAK; 4 Internal Medicine, Jinnah Postgraduate Medical Center, Karachi, PAK

**Keywords:** risk factors, foot amputation, diabetic complication, pakistan, diabetic foot, diabetes mellitus

## Abstract

Introduction

The major grave outcome of diabetic complications is the amputation of lower limb extremities. Recurrent foot infections, trauma, ischemia, and peripheral neuropathy play a crucial role in predicting foot amputation. The aim of this study is to identify the risk factors of diabetic foot amputations in Pakistani patients.

Methods

Patients admitted with diabetic foot-related complications were followed throughout their hospital stay. Their sociodemographic and disease-related characteristics were recorded. Patients who were advised foot amputation were taken as group A, and patients who were managed conservatively were termed as group B. Their characteristics were then compared.

Results

Out of 226 study participants, there were 51 (22.5%) patients in group A who were advised foot amputation. There were more men in group A as compared to group B (72.5% vs. 30.8%; p<0.00001). Group A also had a longer duration of diabetes (15.23 ± 8.52 years vs. 11.98 ± 9.69; p=0.03). Group B included more patients taking insulin therapy (44.5% vs. 37.3%; p=0.002). All three risk factors of atherosclerosis - smoking, hyperlipidemia, and hypertension - were significantly associated with group A (p≤0.05). This coexistence of diabetic nephropathy and retinopathy were more common in group A (p≤0.05).

Conclusion

The incidence of foot amputation in diabetic patients is high. Crucial risk factors include male gender, smoking, hyperlipidemia, hypertension, cardiac history, and the coexistence of diabetic nephropathy and retinopathy.

## Introduction

Globally, there are more than 366-million people suffering from diabetes and this number is projected to increase to half a billion by 2030 [1]. Among many other complications, with a lifetime risk as high as 25%, diabetic foot ulcers (DFU) are a major complication and the pathogenesis involves multiple factors. The primary causative factor involved in diabetic foot ulcer is peripheral neuropathy. Other factors that are responsible for diabetic foot infections are peripheral vascular disease, repetitive trauma, and super-imposing foot infections [2-3]. Infected diabetic foot increases healthcare burden by prolonging hospital admission and increasing antibiotic burden. It is responsible for more than 90% of non-traumatic lower-limb amputations (LLAs) [4-5], which estimates more than a million foot amputations in one year [6-8]. It is associated with a high mortality rate [9].

The risk factor for diabetic foot amputation includes nephropathy, ischemic diabetic foot, and first fasting blood glucose >200 mg/dl [10]. Diabetic nephropathy and peripheral ischemia have been consistently reported as a risk factor for foot amputation [11-12] Other less obvious risk factors include gender, recurrent foot infections, ulcers, abscesses, osteomyelitis, and diabetic retinopathy. Chaturvedi et al. [13] also suggested elevated levels of blood glucose and triglyceride and the presence of retinopathy as key risk factors for amputation.

Considering there is very little data available in Pakistan regarding the risk factors of lower limb amputation in diabetes, this study was conducted to identify those factors. These causative factors will help develop a strategy to prevent this drastic measure and reduce personal as well as the healthcare-associated burden.

## Materials and methods

In this prospective, observational study, individuals admitted in the endocrinology department of a tertiary care institute with diabetic foot lesions and related complications were enrolled after informed consent. Only patients with type II diabetes and age greater than 18 years were included. The study duration was of six months (July-December 2018). The study was approved by the institutional review board and patient consent was attained.

Patients who agreed to participate in the study were followed throughout their hospital stay. Neurovascular examination of lower extremities was conducted. Type and extent of foot lesions were identified. A patient was considered to have ischemic diabetic foot if distal pulses were absent on foot examination and were classified as neuropathic if there was loss or decrease of vibration sense in the foot with a monofilament. Biochemical markers, including random blood glucose (PBG), serum lipid profile, and detailed urine analysis, were evaluated. Patients were classified as having nephropathy when persistent albuminuria was 300 mg or more in 24-hour urine collection. Retinopathy was diagnosed on fundus examination.

Other variables taken into consideration included patient gender, age, duration of diabetes, diabetes treatment, and atherosclerosis risk factors (smoking, hypertension, and hyperlipidemia). Cardiac co-morbidity status was also recorded. There were two disease outcomes in this study, and the patients were divided into two groups accordingly. Group A included patients in whom amputation was advised and group B included patients in whom amputation was not performed. Data was entered and analyzed using SPSS Version 22.0 (IBMCorp, Armonk, NY, US). Mean and standard deviation (SD) were calculated for continuous variables. Frequencies and percentages were calculated for categorical values. Patient factors were compared for both groups and chi-square was applied to find any correlation. P-value≤0.05 was taken as significant.

## Results

Two-hundred and twenty-six type II diabetes patients with foot lesions completed the study. There were 124 men and 102 women in the study. Their mean age was 59.14 ± 9.68 years and their mean duration of diabetes was 8.71 ± 4.77 years.

There were 51 (22.5%) patients in group A who were advised foot amputation and 175 (77.4%) were managed conservatively. In group A, three patients refused amputation and left against medical advice, however, their characteristics were included in this analysis.

There were more men in group A as compared to group B (72.5% vs. 30.8%; p<0.00001). Group A also had a longer duration of diabetes than group B (15.23 ± 8.52 years vs. 11.98 ± 9.69; p=0.03). When patients were compared for their treatment, it was seen that group B included more people using insulin therapy as compared to group A (44.5% vs. 37.3%; p=0.002). As far as oral anti-diabetic therapy was concerned, there were more such individuals in group A as compared to group B (49.0% vs. 33.1%; p=0.004). All three risk factors of atherosclerosis - smoking, hyperlipidemia, and hypertension - were significantly associated with group A (p≤0.05). When co-morbidity status was compared, it was seen that a history of ischemic heart disease (IHD), cerebrovascular stroke, and diabetic nephropathy and retinopathy were more common in group A (p≤0.05). All characteristics are shown in Table 1.

**Table 1:** Comparison of profiles of patients in Group A and group B Abbreviations: CABG, Coronary Artery Bypass Grafting; IHD, Ischemic Heart Disease

Variables	Group A – Amputation Group (N=51)	Group B – Non-Amputation (N=175)	P-Value
Mean age in years	58.60 ± 11.12	57.24 ± 11.18	0.44
Gender			
Male	37 (72.5%)	54 (30.8%)	<0.00001
Female	14 (27.5%)	121 (69.1%)	
Diabetes duration (years)	15.23 ± 8.52	11.98 ± 9.69	0.03
Diabetes treatment			
Insulin	19 (37.3%)	78 (44.5%)	0.002
Oral hypoglycemic agents	25 (49.0%)	58 (33.1%)	0.004
Insulin + oral hypoglycemic agent	7 (13.7%)	39 (22.3%)	0.82
Risk of Atherosclerosis			
Smoking	31 (60.8%)	28 (16.0%)	<0.00001
Hyperlipidemia	40 (78.4%)	26 (14.8%)	<0.0001
Hypertension	25 (49.0%)	36 (20.5%)	0.0005
Co-morbidities (%)			
History of CABG	8 (15.7%)	13 (7.4%)	0.07
History of IHD	19 (37.2%)	35 (20.0%)	0.01
History of cerebrovascular stroke	9 (17.6%)	3 (1.7%)	<0.0001
Diabetic nephropathy	29 (56.8%)	14 (8.0%)	<0.0001
Diabetic retinopathy	15 (29.4%)	23 (13.1%)	0.006
Type of diabetic foot lesion (%)			
Ischemic	34 (66.7%)	78 (44.5%)	0.005
Neuropathic	12 (23.5%)	81 (46.3%)	0.003
Ischemic and neuropathic	5 (9.8%)	16 (9.1%)	0.88

## Discussion

The incidence of foot amputation is high in this study. In this study, male gender, longer duration of disease, oral anti-diabetic therapy, smoking, hyperlipidemia, hypertension, and history of IHD, cerebrovascular stroke, peripheral ischemia, and diabetic nephropathy, and retinopathy were strongly associated with the incidence of foot amputation. Insulin therapy and peripheral neuropathy were related to the non-amputation group in this study.

Knowledge of these factors is important to allow multidisciplinary teams to develop management and treatment protocols. While this study adds to the very scarce data available on the topic, it has limitations. Not all risk factors were taken into account. The study was conducted on hospitalized patients only, which means that the foot lesions are severe enough to be considered for amputation. The incidence of foot amputation might be lower if all other cases of foot lesions, including the ones managed conservatively in the outpatient department are included in the analysis.

Male gender has been repeatedly identified as a risk factor for diabetic foot amputation in other literature [14-15]. Patients on oral hypoglycaemic therapy were more associated with amputation than those taking insulin in this study. However, in another major study, metformin was shown to be associated with 30% of amputation in comparison to insulin, which was associated with 64% of lower extremity amputations [16]. Smoking was deduced as a risk factor in this study. Similarly, in other studies, smoking was shown to be associated with diabetic foot amputation. It has also been shown that smoking cessation may serve as a protective measure against diabetic foot amputation [17]. Hypertension is a known contributor to the development and progression of the complications of diabetes [18].

This study established hypertension as a risk factor for foot amputation. This link was established in other literature as well [19-20]. Similar is the case with hyperlipidemia [20]. These factors predispose to atherosclerosis, which increases underlying peripheral ischemia. Diabetic patients have a high risk of atherosclerotic peripheral vascular disease. Without doubt, because of peripheral vascular disease, there will be an obstruction in blood flow, which eventually will make it difficult for healing. Ischemic diabetic foot, in turn, results in gangrenous lesions. Limb ischemia has also been identified as a risk factor for amputation in patients with diabetic foot lesions. Calle-Pascual, in his study, showed that all major amputations patients had peripheral vascular disease [21]. This study established nephropathy as a risk factor for foot amputation. The connecting link between limb amputation and diabetic nephropathy is also peripheral ischemia. The likelihood of peripheral arterial disease increases with decreasing creatinine clearance. In patients with both diabetes and chronic kidney disease, the risk of foot amputation increases by two to six times as compared to those who have diabetes only [22].

Diabetic foot lesions are responsible for health and socioeconomic issues, have adverse effects on the quality of life, and pose a serious economic burden on healthcare. Foot amputations are completely preventable. Early recognition of high-risk patients, optimum control of patient factors, and a multidisciplinary approach toward the management of foot-related lesions in diabetes can help in reducing the incidence of foot amputation.

## Conclusions

The incidence of foot amputation in diabetic patients is high. Crucial risk factors include male gender, smoking, hyperlipidemia, hypertension, cardiac history, and the coexistence of diabetic nephropathy and retinopathy. Lower extremity amputation in diabetes is a preventable complication. It requires individual patient care, early recognition of risk factors, and a multidisciplinary approach to manage diabetic foot lesions. Identifying risk factors will help in providing high-risk patients with the right care for early wound healing and for preventing any worse outcome.
